# Supplementary material for: A Novel Streptococcus thermophilus FUA329 Isolated from Human Breast Milk Capable of Producing Urolithin A from Ellagic Acid
Source: Foods. 2022 Oct 20;11(20):3280. doi: 10.3390/foods11203280 (PMC9601659; doi:10.3390/foods11203280)
Supplement: Supplementary file 1 [file foods-11-03280-s001.zip › foods-1938121-supplementary.pdf]

Supplementary Materials

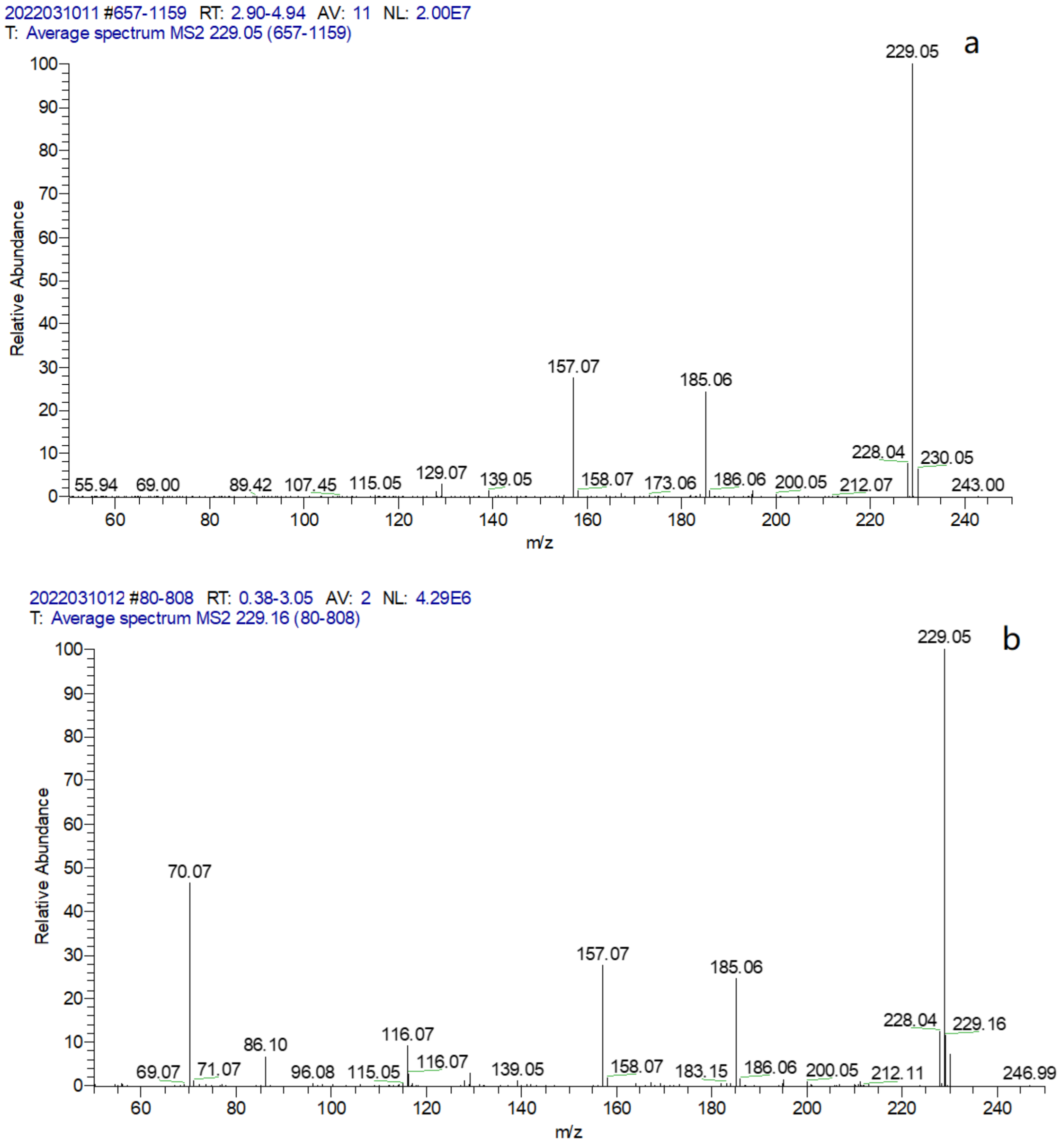

Supplementary Figure S1. HPLC-MS/MS analysis: (**a**) The standard urolithin A; (**b**) The urolithin A of the fermentation broth of the strain FUA329.

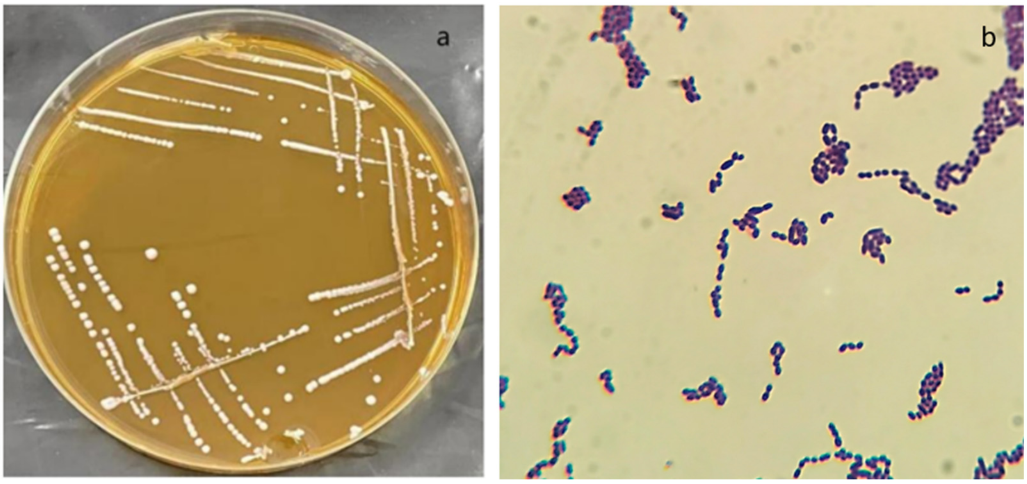

Supplementary Figure S2. (**a**) The colony morphology of the strain FUA329 on the ABB plate; (**b**) light microscopic picture (10× 100 magnification).
